# COVID-19 and comedications in atrial fibrillation—a case–control study in Stockholm

**DOI:** 10.1007/s10654-023-00967-9

**Published:** 2023-01-28

**Authors:** Max Bell, Anders Ekbom, Marie Linder

**Affiliations:** 1 Department of Perioperative Medicine and Intensive Care, Karolinska University Hospital, 171 76 Stockholm, Sweden; 2 Department of Physiology and Pharmacology, Karolinska Institutet, Stockholm, Sweden; 3 Department of Medicine, Centre for Pharmacoepidemiology, Karolinska Institutet, Karolinska University Hospital Solna, T2, 171 76 Stockholm, Sweden

**Keywords:** Anticoagulants, Communicable diseases, COVID-19, Epidemiology

## Abstract

To test the main hypothesis that anticoagulation reduces risk of hospitalization, intensive care unit (ICU) admission and death in COVID-19. Nested case–control study among patients with atrial fibrillation (AF) in Stockholm. COVID-19 cases were matched to five disease-free controls with same sex, born within ± 1 years. Source population was individuals in Stockholm with AF 1997–2020. Swedish regional and national registers are used. National registers cover hospitals and outpatient clinics, local registers cover primary care. Records were linked through the personal identity number assigned to each Swedish resident. Cases were individuals with COVID-19 (diagnosis, ICU admission, or death). The AF source population consisted of 179,381 individuals from which 7548 cases were identified together with 37,145 controls. The number of cases (controls) identified from hospitalization, ICU admission or death were 5916 (29,035), 160 (750) and 1472 (7,360). The proportion of women was 40% for hospitalization and death, but 20% and 30% for admission to ICU in wave one and two, respectively. Primary outcome was mortality, secondary outcome was hospitalization, tertiary outcome was ICU admission, all with COVID-19. Odds ratios (95% confidence interval) for antithrombotics were 0.79 (0.66–0.95) for the first wave and 0.80 (0.64–1.01) for the second wave. Use of anticoagulation among patients with arrythmias infected with COVID-19 is associated with lower risk of hospitalization and death. If further COVID-variants emerge, or other infections with prothrombotic properties, this emphasize need for physicians to ensure compliance among vulnerable patients.

**Supplementary Information:**

The online version contains supplementary material available at 10.1007/s10654-023-00967-9.

## Introduction

Severe acute respiratory syndrome coronavirus 2 (SARS-CoV-2) causes Coronavirus disease 2019 (COVID-19). Since 31 December 2019 until today (December 2022) over 650 million cases and approximately 6.5 million deaths have been reported and it continues to be a global health threat. The Stockholm Region in Sweden was hit hard by the pandemic with a surge in cases at the end of March and beginning of April of 2020.

The infection principally causes respiratory symptoms, ranging from intermittent coughing, via dyspnea, to life threatening acute respiratory distress syndrome [1]. Additionally, coagulopathy is a common and dangerous complication, in particular among severe cases of COVID-19 in SARS-CoV-2-infected patients [2, 3]. In critically ill COVID-19 patients, the incidence of thrombotic complications, including pulmonary embolism, is reported to be over 30% [2, 4]. This insight led to changes in how hospitalized COVID-19 patients are treated with regards to thromboprophylaxis. A recent systemic review of international guidance revealed that 8/10 documents recommended thromboprophylaxis for all patients, and that intermediate or therapeutic doses of low molecular weight heparin (LMWH) should be given to patients with elevated risk of venous thromboembolism (VTE) [5]. Notably, these recommendations exist despite limited evidence. In studies comparing different treatment strategies, results vary. One randomized controlled, adaptive, open label clinical trial of 465 hospitalized adults compared therapeutic with prophylactic dose heparin (LMWH or unfractionated heparin), there was no difference in their primary composite outcome, but with slightly lower odds of death at 30 days [6]. In a propensity score-matched cohort study on COVID-19 patients in medical wards, intermediate-dose prophylactic anticoagulation (AC) compared with standard-dose prophylactic AC did not detect any difference in in-hospital mortality [7]. Finally, in a US study, Kuno et al. found that in-hospital mortality was not significantly different in patients with anticoagulation before admission compared to those without [8].

Naturally, speculation as to if pre-medication with oral anticoagulants (OAC) is beneficial for patients later infected with SARS-CoV-2 has been prevalent. Results from investigations vary, the Lean European Open Survey on SARS-CoV-2 Infected Patients (LEOSS) registry found oral anticoagulants to be associated with lower risk of death and other outcomes [9]. In contrast, a large Swedish registry study did not find ongoing OAC use to reduce risk of severe COVID-19 [10]. One problem with most previous studies of anticoagulant therapy is that they are hampered by confounding; patients with anticoagulation therapy are in general sicker than patients without the same medication which will affect outcomes especially death and/or severity of disease. This investigation addresses the issue of confounding by restricting the study base to patients with atrial fibrillation to remove the effect of their cardiovascular disease using a very high-resolution dataset from Stockholm. Moreover, ICU admission in Stockholm during the first wave was prone to triage/patient selection due to the massive surge of patients. This issue is addressed by reporting results by wave.

Also, diabetes/antidiabetics [11–13], cardiovascular disease and its treatment [14], sex steroids [15, 16], corticosteroids [17] and hypertension/antihypertensives [18, 19] have been identified as factors affecting the outcome of COVID-19 infection.

In patients with pre-existing atrial fibrillation (AF) we performed a nested case–control study, testing the main hypothesis that oral anticoagulation as a class would confer a reduced risk of COVID-19 as measured by hospitalization, ICU admission or death in individuals with AF. We also tested secondary hypotheses of altered COVID-19 risks for other drug classes and comorbidities. All hypotheses were tested pooled and by wave for the first two COVID-19 waves in Sweden.

## Methods

### Study design and data sources

A case–control study design was chosen since it allows for investigation of several risk factors and is suitable for establishing associations within new fields [20]. This nested case–control study used various Swedish regional and national health registers as data sources covering data until March 2021.

*The Stockholm regional healthcare data warehouse (VAL-database)*, containing information from both hospitals and outpatient clinics on preexisting diagnoses according to the International Classification of Diseases (ICD) and Swedish codes (KVÅ) for procedures.

*The Swedish Prescribed Drug Register (PDR)* on filled prescriptions coded by anatomical therapeutic classification (ATC) codes [21].

*The Swedish National Patient register (NPR)*, was used in addition to the VAL-database. It too has information from hospitals and outpatient clinics, with ICD codes regarding preexisting diagnoses and KVÅ-codes for procedures.

*The Swedish Cause of Death Register (SCR)* allowed us to identify COVID-19 related deaths [22].

*The Swedish intensive care register (SIR)* was used to track ICU admissions.

*SmiNet* [23]—a national electronic surveillance system for reporting of communicable diseases—has information on infection with COVID-19, since February 1, 2020, and it is mandatory for all Swedish laboratories to report findings of COVID-19 to SmiNet.

Registers and records were linked through the unique personal identity number assigned to each Swedish resident at birth or immigration [24].

### Study population

The source population (nest) consisted of all individuals with a recorded diagnosis of atrial fibrillation (ICD10: I48, including paroxysmal atrial fibrillation, persistent atrial fibrillation, chronic atrial fibrillation, typical atrial flutter, atypical atrial flutter, and unspecified atrial fibrillation/flutter) between January 1st 1997 and December 31st 2020 residing in the Stockholm region.

### Exposure, cases and controls

All individuals recorded with a diagnosis of COVID-19 (ICD10: U071, U072, U109) or a COVID-19 related procedure (KVÅ: ZV100) together with either actions concerning notifiable communicable diseases (KVÅ: AV097, DV091, GD001), admission to inpatient care (KVÅ: XS100) or oxygen treatment (KVÅ: DG009, DG015, DG028, DV028, QD014) in NPR or the VAL-database, recorded as treated in intensive care for COVID-19 in SIR or recorded as dead with COVID-19 (U071, U072, U109) as underlying or contributory cause in SCR were selected as cases for hospitalization, ICU admission or death, respectively.

For COVID-19 hospitalization the first date retrieved from NPR or the VAL-database was used as index date. For COVID-19 in intensive care the first date recorded in SIR was identified as index date. For COVID-19 deaths the date of death was the index date. Only cases with a history of AF were included, i.e. only cases who had an AF-diagnosis recorded *before* their index date.

Controls were matched to cases by sex and age. Up to five controls [25] per case were sampled with replacement from the source population with the same sex as the case, born within ± 1 years of the case, alive, with a history of AF and without recorded signs of COVID-19 at the index date of the case. Each outcome, hospitalization, ICU admission or death, were matched separately.

### Outcome

The primary outcome was mortality with confirmed COVID-19 infection. The secondary outcome was hospitalization, and the tertiary outcome was intensive care unit (ICU) admission with confirmed COVID-19 infection.

### Exposure

Exposure was defined via ATC-codes from filled prescriptions. The included exposures were the drug classes (ATC-code): drugs used in diabetes (A10), antithrombotic agents (B01), cardiac therapy (C01), antihypertensives (C02), diuretics (C03), beta blocking agents (C07), calcium channel blockers (C08), agents acting on the renin-angiotensin system (C09), lipid modifying agents (C10), sex hormones and modulators of the genital system (G03), corticosteroids for systemic use (H02), and endocrine therapy (L02). For a complete list of drugs included in each class, see Supplementary table 1. An individual was assumed to be exposed to a drug class if they had supply of a drug within that class at the index date. Supply was assessed from number of filled defined daily doses (DDDs) plus a grace period of 30 days for the prescription filled closest before the index date. The DDD is defined by WHO as the assumed average maintenance dose per day for a drug used for its main indication in adults [26].

### Statistical analysis

Each outcome (mortality, hospitalization and ICU admission) was modelled separately by two COVID-19 waves and pooled using conditional logistic regression adjusted for all exposures and identified confounders. The COVID-19 waves were defined as first wave between January 1st 2020 and August 31st 2020, second wave between September 1st 2020 and March 31st 2021. It was important to analyze the two pandemic waves separately due to the massive surge during the first one. Confounders included: ischemic heart disease, heart failure/cardiomyopathy, valve disorder, ischemic stroke/ transient ischemic attack (TIA)/systemic thromboembolism, hemorrhagic/unspecified stroke, other vascular disease, arrhythmia (other than AF/flutter), lung disease, renal disease, liver disease, venous thromboembolism, and malignancies [10]. See Supplementary table 2 for ICD-10 and KVÅ codes used for defining the covariates. All analyses were performed using SAS software version 9.4 (SAS Institute Inc., Cary, NC, USA).

### Patient and public involvement

No patients were actively involved or asked for advice in the current study.

## Results

**Fig. 1:**
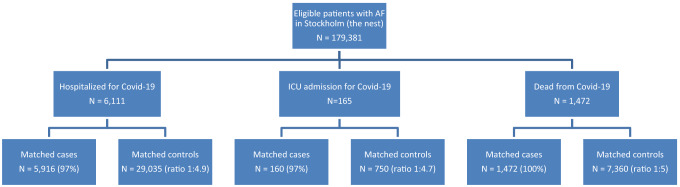
Flowchart illustrating the source population (nest) of patients with atrial fibrillation (AF) in Stockholm and the identification of COVID-19 cases (hospitalized, ICU admitted or dead) and the subsequent matching of COVID-19-free controls

The source population consisted of 179,381 individuals from which 7548 cases with COVID-19 overall were identified together with 37,145 matched controls. The number of included cases (controls) for hospital admission, ICU admission and death were 5916 (29,035), 160 (750) and 1472 (7360), respectively. Figure 1 illustrates the inclusion of cases and controls, and Table 1 shows baseline data.

**Table 1:** Baseline characteristics, number (proportion) of individuals by COVID-19 wave and pooled, for COVID-19 cases and their COVID-19-free matched controls, for each COVID-19 outcome (hospitalization, ICU admission and death)

Diagnosis or treatment subgroup	Value	Wave^a^	Outcome: Hospitalized for COVID-19		Outcome: ICU admitted for COVID-19		Outcome: Dead from COVID-19	
			Controls without the outcome	Cases with the outcome	Controls without the outcome	Cases with the outcome	Controls without the outcome	Cases with the outcome
Overall		1	18,396 (100%)	3754 (100%)	601 (100%)	130 (100%)	4435 (100%)	887 (100%)
		2	10,639 (100%)	2162 (100%)	149 (100%)	30 (100%)	2925 (100%)	585 (100%)
		Pooled	29,035 (100%)	5916 (100%)	750 (100%)	160 (100%)	7360 (100%)	1472 (100%)
Sex	Female	1	7834 (42.6%)	1604 (42.7%)	122 (20.3%)	28 (21.5%)	1870 (42.2%)	374 (42.2%)
	Male	1	10,562 (57.4%)	2150 (57.3%)	479 (79.7%)	102 (78.5%)	2565 (57.8%)	513 (57.8%)
	Female	2	4595 (43.2%)	938 (43.4%)	44 (29.5%)	9 (30.0%)	1250 (42.7%)	250 (42.7%)
	Male	2	6044 (56.8%)	1224 (56.6%)	105 (70.5%)	21 (70.0%)	1675 (57.3%)	335 (57.3%)
	Female	Pooled	12,429 (42.8%)	2542 (43.0%)	166 (22.1%)	37 (23.1%)	3120 (42.4%)	624 (42.4%)
	Male	Pooled	16,606 (57.2%)	3374 (57.0%)	584 (77.9%)	123 (76.9%)	4240 (57.6%)	848 (57.6%)
Age	0–9	1	40 (0.2%)	8 (0.2%)	0 (0.0%)	0 (0.0%)	0 (0.0%)	0 (0.0%)
	10–19	1	0 (0.0%)	0 (0.0%)	0 (0.0%)	0 (0.0%)	0 (0.0%)	0 (0.0%)
	20–29	1	18 (0.1%)	6 (0.2%)	0 (0.0%)	0 (0.0%)	0 (0.0%)	0 (0.0%)
	30–39	1	52 (0.3%)	9 (0.2%)	0 (0.0%)	0 (0.0%)	0 (0.0%)	0 (0.0%)
	40–49	1	245 (1.3%)	49 (1.3%)	27 (4.5%)	6 (4.6%)	0 (0.0%)	0 (0.0%)
	50–59	1	784 (4.3%)	164 (4.4%)	71 (11.8%)	17 (13.1%)	35 (0.8%)	7 (0.8%)
	60–69	1	1946 (10.6%)	396 (10.5%)	207 (34.4%)	47 (36.2%)	160 (3.6%)	33 (3.7%)
	70–79	1	5301 (28.8%)	1069 (28.5%)	224 (37.3%)	44 (33.8%)	730 (16.5%)	143 (16.1%)
	80–89	1	6826 (37.1%)	1399 (37.3%)	61 (10.1%)	13 (10.0%)	1936 (43.7%)	382 (43.1%)
	90 +	1	3184 (17.3%)	654 (17.4%)	11 (1.8%)	3 (2.3%)	1574 (35.5%)	322 (36.3%)
Age	0–9	2	25 (0.2%)	5 (0.2%)	0 (0.0%)	0 (0.0%)	0 (0.0%)	0 (0.0%)
	10–19	2	0 (0.0%)	0 (0.0%)	0 (0.0%)	0 (0.0%)	0 (0.0%)	0 (0.0%)
	20–29	2	0 (0.0%)	0 (0.0%)	0 (0.0%)	0 (0.0%)	0 (0.0%)	0 (0.0%)
	30–39	2	28 (0.3%)	6 (0.3%)	0 (0.0%)	0 (0.0%)	0 (0.0%)	0 (0.0%)
	40–49	2	139 (1.3%)	30 (1.4%)	9 (6.0%)	2 (6.7%)	0 (0.0%)	0 (0.0%)
	50–59	2	492 (4.6%)	98 (4.5%)	13 (8.7%)	2 (6.7%)	11 (0.4%)	2 (0.3%)
	60–69	2	1022 (9.6%)	204 (9.4%)	47 (31.5%)	10 (33.3%)	77 (2.6%)	16 (2.7%)
	70–79	2	3150 (29.6%)	636 (29.4%)	32 (21.5%)	7 (23.3%)	560 (19.1%)	109 (18.6%)
	80–89	2	4076 (38.3%)	841 (38.9%)	43 (28.9%)	8 (26.7%)	1299 (44.4%)	268 (45.8%)
	90 +	2	1707 (16.0%)	342 (15.8%)	5 (3.4%)	1 (3.3%)	978 (33.4%)	190 (32.5%)
Age	0–9	Pooled	65 (0.2%)	13 (0.2%)	0 (0.0%)	0 (0.0%)	0 (0.0%)	0 (0.0%)
	10–19	Pooled	0 (0.0%)	0 (0.0%)	0 (0.0%)	0 (0.0%)	0 (0.0%)	0 (0.0%)
	20–29	Pooled	18 (0.1%)	6 (0.1%)	0 (0.0%)	0 (0.0%)	0 (0.0%)	0 (0.0%)
	30–39	Pooled	80 (0.3%)	15 (0.3%)	0 (0.0%)	0 (0.0%)	0 (0.0%)	0 (0.0%)
	40–49	Pooled	384 (1.3%)	79 (1.3%)	36 (4.8%)	8 (5.0%)	0 (0.0%)	0 (0.0%)
	50–59	Pooled	1276 (4.4%)	262 (4.4%)	84 (11.2%)	19 (11.9%)	46 (0.6%)	9 (0.6%)
	60–69	Pooled	2968 (10.2%)	600 (10.1%)	254 (33.9%)	57 (35.6%)	237 (3.2%)	49 (3.3%)
	70–79	Pooled	8451 (29.1%)	1705 (28.8%)	256 (34.1%)	51 (31.9%)	1290 (17.5%)	252 (17.1%)
	80–89	Pooled	10,902 (37.5%)	2240 (37.9%)	104 (13.9%)	21 (13.1%)	3235 (44.0%)	650 (44.2%)
	90 +	Pooled	4891 (16.8%)	996 (16.8%)	16 (2.1%)	4 (2.5%)	2552 (34.7%)	512 (34.8%)
Arrhythmia, other than AF	Yes	1	4528 (25%)	901 (24%)	154 (26%)	17 (13%)	1080 (24%)	221 (25%)
		2	2591 (24%)	527 (24%)	34 (23%)	6 (20%)	750 (26%)	146 (25%)
		Pooled	7119 (25%)	1428 (24%)	188 (25%)	23 (14%)	1830 (25%)	367 (25%)
Bleeding Stroke	Yes	1	695 (4%)	204 (5%)	9 (1%)	2 (2%)	205 (5%)	81 (9%)
		2	424 (4%)	127 (6%)	3 (2%)	0 (0%)	119 (4%)	46 (8%)
		Pooled	1119 (4%)	331 (6%)	12 (2%)	2 (1%)	324 (4%)	127 (9%)
Cancer	Yes	1	1997 (11%)	569 (15%)	49 (8%)	17 (13%)	551 (12%)	141 (16%)
		2	1175 (11%)	317 (15%)	17 (11%)	2 (7%)	352 (12%)	107 (18%)
		Pooled	3172 (11%)	886 (15%)	66 (9%)	19 (12%)	903 (12%)	248 (17%)
Heart Failure	Yes	1	6513 (35%)	1861 (50%)	169 (28%)	33 (25%)	1886 (43%)	528 (60%)
		2	3649 (34%)	1038 (48%)	41 (28%)	8 (27%)	1182 (40%)	366 (63%)
		Pooled	10,162 (35%)	2899 (49%)	210 (28%)	41 (26%)	3068 (42%)	894 (61%)
Ischemic heart disease	Yes	1	4860 (26%)	1282 (34%)	115 (19%)	27 (21%)	1354 (31%)	327 (37%)
		2	2800 (26%)	737 (34%)	29 (19%)	9 (30%)	874 (30%)	242 (41%)
		Pooled	7660 (26%)	2019 (34%)	144 (19%)	36 (23%)	2228 (30%)	569 (39%)
Ischemic Stroke	Yes	1	2602 (14%)	745 (20%)	46 (8%)	9 (7%)	735 (17%)	225 (25%)
		2	1472 (14%)	439 (20%)	20 (13%)	2 (7%)	508 (17%)	172 (29%)
		Pooled	4074 (14%)	1184 (20%)	66 (9%)	11 (7%)	1243 (17%)	397 (27%)
Liver disease	Yes	1	417 (2%)	174 (5%)	19 (3%)	6 (5%)	62 (1%)	20 (2%)
		2	226 (2%)	91 (4%)	6 (4%)	3 (10%)	45 (2%)	26 (4%)
		Pooled	643 (2%)	265 (4%)	25 (3%)	9 (6%)	107 (1%)	46 (3%)
Lung disease	Yes	1	3835 (21%)	1222 (33%)	110 (18%)	32 (25%)	915 (21%)	261 (29%)
		2	2180 (20%)	670 (31%)	29 (19%)	5 (17%)	627 (21%)	206 (35%)
		Pooled	6015 (21%)	1892 (32%)	139 (19%)	37 (23%)	1542 (21%)	467 (32%)
Other Vascular disease	Yes	1	1883 (10%)	607 (16%)	56 (9%)	7 (5%)	502 (11%)	145 (16%)
		2	1052 (10%)	307 (14%)	12 (8%)	3 (10%)	316 (11%)	106 (18%)
		Pooled	2935 (10%)	914 (15%)	68 (9%)	10 (6%)	818 (11%)	251 (17%)
Renal disease	Yes	1	2533 (14%)	1031 (27%)	63 (10%)	34 (26%)	808 (18%)	370 (42%)
		2	1473 (14%)	561 (26%)	18 (12%)	5 (17%)	478 (16%)	241 (41%)
		Pooled	4006 (14%)	1592 (27%)	81 (11%)	39 (24%)	1286 (17%)	611 (42%)
Valve disorder	Yes	1	2331 (13%)	628 (17%)	56 (9%)	9 (7%)	642 (14%)	151 (17%)
		2	1391 (13%)	336 (16%)	13 (9%)	3 (10%)	382 (13%)	96 (16%)
		Pooled	3722 (13%)	964 (16%)	69 (9%)	12 (8%)	1024 (14%)	247 (17%)
Venous Thrombo-embolism	Yes	1	1666 (9%)	485 (13%)	43 (7%)	21 (16%)	443 (10%)	144 (16%)
		2	947 (9%)	268 (12%)	11 (7%)	3 (10%)	284 (10%)	89 (15%)
		Pooled	2613 (9%)	753 (13%)	54 (7%)	24 (15%)	727 (10%)	233 (16%)
Insulins and Analogues	Yes	1	2263 (12%)	687 (18%)	79 (13%)	27 (21%)	492 (11%)	139 (16%)
		2	1262 (12%)	422 (20%)	18 (12%)	6 (20%)	293 (10%)	98 (17%)
		Pooled	3525 (12%)	1109 (19%)	97 (13%)	33 (21%)	785 (11%)	237 (16%)
Antithrombotic Agents	Yes	1	13,653 (74%)	2615 (70%)	407 (68%)	58 (45%)	3386 (76%)	633 (71%)
		2	7855 (74%)	1570 (73%)	94 (63%)	21 (70%)	2090 (71%)	377 (64%)
		Pooled	21,508 (74%)	4185 (71%)	501 (67%)	79 (49%)	5476 (74%)	1010 (69%)
Cardiac Therapy	Yes	1	3002 (16%)	694 (18%)	88 (15%)	9 (7%)	725 (16%)	175 (20%)
		2	1740 (16%)	414 (19%)	27 (18%)	6 (20%)	488 (17%)	93 (16%)
		Pooled	4742 (16%)	1108 (19%)	115 (15%)	15 (9%)	1213 (16%)	268 (18%)
Antihypertensives	Yes	1	201 (1%)	52 (1%)	8 (1%)	1 (1%)	54 (1%)	10 (1%)
		2	121 (1%)	37 (2%)	2 (1%)	1 (3%)	17 (1%)	8 (1%)
		Pooled	322 (1%)	89 (2%)	10 (1%)	2 (1%)	71 (1%)	18 (1%)
Diuretics	Yes	1	5851 (32%)	1658 (44%)	121 (20%)	24 (18%)	1761 (40%)	469 (53%)
		2	3344 (31%)	947 (44%)	31 (21%)	6 (20%)	1001 (34%)	315 (54%)
		Pooled	9195 (32%)	2605 (44%)	152 (20%)	30 (19%)	2762 (38%)	784 (53%)
Beta Blocking Agents	Yes	1	10,196 (55%)	2161 (58%)	339 (56%)	49 (38%)	2533 (57%)	540 (61%)
		2	5726 (54%)	1262 (58%)	74 (50%)	15 (50%)	1470 (50%)	297 (51%)
		Pooled	15,922 (55%)	3423 (58%)	413 (55%)	64 (40%)	4003 (54%)	837 (57%)
Calcium Channel Blockers	Yes	1	4351 (24%)	894 (24%)	121 (20%)	37 (28%)	1016 (23%)	183 (21%)
		2	2492 (23%)	549 (25%)	28 (19%)	10 (33%)	659 (23%)	99 (17%)
		Pooled	6843 (24%)	1443 (24%)	149 (20%)	47 (29%)	1675 (23%)	282 (19%)
Agents Acting on the Renin-Angiotensin System	Yes	1	9519 (52%)	1883 (50%)	302 (50%)	55 (42%)	2221 (50%)	396 (45%)
		2	5426 (51%)	1138 (53%)	75 (50%)	16 (53%)	1414 (48%)	248 (42%)
		Pooled	14,945 (51%)	3021 (51%)	377 (50%)	71 (44%)	3635 (49%)	644 (44%)
Lipid Modifying Agents	Yes	1	6541 (36%)	1430 (38%)	220 (37%)	38 (29%)	1466 (33%)	275 (31%)
		2	3850 (36%)	880 (41%)	64 (43%)	10 (33%)	1008 (34%)	172 (29%)
		Pooled	10,391 (36%)	2310 (39%)	284 (38%)	48 (30%)	2474 (34%)	447 (30%)
Sex Hormones and Modulators of the Genital System	Yes	1	691 (4%)	138 (4%)	18 (3%)	2 (2%)	146 (3%)	20 (2%)
		2	414 (4%)	77 (4%)	7 (5%)	2 (7%)	93 (3%)	3 (1%)
		Pooled	1105 (4%)	215 (4%)	25 (3%)	4 (3%)	239 (3%)	23 (2%)
Corticosteroids for Systemic Use	Yes	1	913 (5%)	438 (12%)	25 (4%)	11 (8%)	252 (6%)	93 (10%)
		2	501 (5%)	216 (10%)	10 (7%)	2 (7%)	159 (5%)	79 (14%)
		Pooled	1414 (5%)	654 (11%)	35 (5%)	13 (8%)	411 (6%)	172 (12%)
Endocrine Therapy	Yes	1	561 (3%)	157 (4%)	16 (3%)	1 (1%)	176 (4%)	34 (4%)
		2	348 (3%)	72 (3%)	3 (2%)	0 (0%)	123 (4%)	25 (4%)
		Pooled	909 (3%)	229 (4%)	19 (3%)	1 (1%)	299 (4%)	59 (4%)

^a^The 1st wave covers 2020–01-01—2020–08-31, the 2nd wave covers 2020–09-01—2020–12-31, pooled means the two waves together

Table 2 details the odds ratios (ORs) and confidence intervals (95% CI) pooled and for the first and second wave of COVID-19 in Stockholm, with respect to exposure of pre-medication. Several drugs are associated to lower odds of hospital admission and death. Antithrombotic drugs stand out, as they were associated with low odds of hospitalization and death both during the first and second wave, with all results, except for death during the second wave, being statistically significant. The finding that lipid lowering agents have low ORs for death has been shown previously [27].

**Table 2:** Odds ratios with 95% confidence intervals for exposures for each outcome, hospitalization, ICU admission and death, by COVID-19 wave and pooled

Exposure	Wave*	Hospitalized		ICU		Death	
		Crude	Adjusted^a^	Crude	Adjusted^a^	Crude	Adjusted^a^
Insulins and analogues	1	1.63 (1.48–1.79)	1.41 (1.27–1.56)	1.86 (1.13–3.06)	2.39 (1.25–4.58)	1.49 (1.22–1.83)	1.34 (1.07–1.68)
	2	1.84 (1.62–2.08)	1.54 (1.35–1.77)	1.82 (0.64–5.16)	1.64 (0.42–6.49)	1.83 (1.42–2.35)	1.58 (1.17–2.13)
	Pooled	1.44 (1.36–1.54)	1.32 (1.23–1.40)	1.70 (1.58–1.83)	1.46 (1.34–1.58)	1.86 (1.18–2.91)	2.19 (1.26–3.80)
Antithrombotic agents	1	0.79 (0.73–0.85)	0.73 (0.67–0.80)	0.33 (0.21–0.51)	0.35 (0.20–0.62)	0.77 (0.65–0.90)	0.79 (0.66–0.95)
	2	0.94 (0.84–1.04)	0.83 (0.74–0.93)	1.44 (0.58–3.57)	1.47 (0.52–4.16)	0.70 (0.57–0.85)	0.80 (0.64–1.01)
	Pooled	0.90 (0.86–0.95)	0.86 (0.82–0.91)	0.84 (0.79–0.89)	0.76 (0.71–0.82)	0.44 (0.30–0.65)	0.51 (0.32–0.81)
Cardiac therapy	1	1.17 (1.06–1.28)	0.99 (0.90–1.10)	0.45 (0.22–0.91)	0.69 (0.30–1.58)	1.27 (1.05–1.53)	1.07 (0.86–1.32)
	2	1.22 (1.08–1.37)	1.00 (0.88–1.14)	1.13 (0.42–3.08)	1.10 (0.35–3.50)	0.94 (0.74–1.20)	0.80 (0.60–1.06)
	Pooled	1.15 (1.08–1.22)	1.02 (0.96–1.08)	1.18 (1.10–1.27)	0.99 (0.92–1.08)	0.58 (0.33–1.03)	0.81 (0.43–1.53)
Antihypertensives	1	1.29 (0.95–1.75)	0.90 (0.65–1.25)	0.53 (0.06–4.44)	0.11 (0.01–1.16)	0.92 (0.47–1.83)	0.77 (0.37–1.59)
	2	1.54 (1.06–2.23)	1.16 (0.78–1.73)	2.50 (0.23–27.57)	2.04 (0.12–35.48)	2.35 (1.02–5.45)	1.90 (0.72–5.00)
	Pooled	1.20 (0.99–1.46)	0.96 (0.79–1.18)	1.38 (1.09–1.75)	0.99 (0.77–1.28)	0.90 (0.19–4.26)	0.37 (0.06–2.10)
Diuretics	1	1.77 (1.65–1.91)	1.26 (1.15–1.37)	0.89 (0.54–1.48)	0.69 (0.35–1.38)	1.74 (1.50–2.01)	1.18 (0.99–1.40)
	2	1.78 (1.61–1.96)	1.26 (1.12–1.41)	0.95 (0.35–2.56)	1.50 (0.45–5.00)	2.43 (2.01–2.94)	1.78 (1.41–2.25)
	Pooled	1.56 (1.49–1.64)	1.26 (1.20–1.33)	1.78 (1.67–1.88)	1.26 (1.17–1.35)	0.90 (0.58–1.42)	0.75 (0.43–1.31)
Beta blocking agents	1	1.10 (1.02–1.18)	0.96 (0.89–1.04)	0.47 (0.31–0.70)	0.59 (0.36–0.98)	1.17 (1.01–1.36)	1.05 (0.89–1.24)
	2	1.21 (1.10–1.34)	1.01 (0.91–1.12)	1.01 (0.45–2.25)	0.83 (0.28–2.40)	1.02 (0.85–1.23)	0.86 (0.70–1.07)
	Pooled	1.08 (1.03–1.13)	0.98 (0.94–1.03)	1.14 (1.08–1.21)	0.98 (0.92–1.04)	0.54 (0.38–0.77)	0.65 (0.43–1.00)
Calcium channel blockers	1	1.01 (0.93–1.09)	1.05 (0.96–1.15)	1.69 (1.07–2.64)	2.30 (1.26–4.20)	0.87 (0.73–1.04)	0.94 (0.78–1.15)
	2	1.12 (1.00–1.24)	1.13 (1.00–1.26)	2.22 (0.92–5.35)	2.34 (0.77–7.10)	0.70 (0.56–0.89)	0.74 (0.57–0.96)
	Pooled	0.98 (0.93–1.04)	1.01 (0.95–1.06)	1.05 (0.98–1.12)	1.08 (1.00–1.16)	1.78 (1.19–2.66)	2.19 (1.34–3.58)
Agents acting on the renin-angiotensin system	1	0.94 (0.87–1.01)	0.88 (0.82–0.96)	0.74 (0.50–1.09)	0.98 (0.58–1.66)	0.80 (0.69–0.93)	0.79 (0.68–0.93)
	2	1.08 (0.98–1.18)	0.94 (0.85–1.04)	1.14 (0.50–2.58)	0.83 (0.27–2.56)	0.78 (0.65–0.94)	0.82 (0.66–1.01)
	Pooled	0.92 (0.88–0.96)	0.85 (0.81–0.89)	0.99 (0.93–1.05)	0.90 (0.85–0.96)	0.80 (0.56–1.14)	0.92 (0.59–1.45)
Lipid modifying agents	1	1.12 (1.04–1.21)	0.94 (0.87–1.03)	0.74 (0.48–1.14)	0.65 (0.36–1.17)	0.90 (0.77–1.06)	0.76 (0.63–0.92)
	2	1.23 (1.12–1.36)	0.99 (0.88–1.11)	0.63 (0.26–1.54)	0.42 (0.12–1.42)	0.79 (0.65–0.96)	0.61 (0.48–0.78)
	Pooled	1.06 (1.02–1.12)	0.92 (0.87–0.97)	1.16 (1.09–1.23)	0.96 (0.90–1.03)	0.72 (0.49–1.06)	0.57 (0.34–0.96)
Sex hormones and modulators of the genital system	1	0.97 (0.81–1.18)	0.99 (0.81–1.20)	0.48 (0.11–2.15)	0.44 (0.09–2.14)	0.67 (0.42–1.08)	0.69 (0.42–1.15)
	2	0.89 (0.69–1.16)	0.90 (0.69–1.17)	1.47 (0.24–8.85)	1.64 (0.21–13.04)	0.15 (0.05–0.47)	0.18 (0.05–0.58)
	Pooled	0.91 (0.81–1.03)	0.94 (0.84–1.06)	0.95 (0.81–1.10)	0.96 (0.82–1.12)	0.71 (0.23–2.15)	0.70 (0.22–2.24)
Corticosteroids for systemic use	1	2.55 (2.26–2.87)	1.93 (1.69–2.19)	2.01 (0.97–4.17)	2.27 (0.94–5.44)	1.96 (1.52–2.52)	1.49 (1.14–1.96)
	2	2.29 (1.94–2.72)	1.84 (1.54–2.20)	0.98 (0.20–4.71)	0.48 (0.05–4.29)	2.75 (2.06–3.67)	1.95 (1.40–2.72)
	Pooled	1.98 (1.82–2.15)	1.65 (1.51–1.80)	2.46 (2.23–2.71)	1.89 (1.70–2.10)	1.73 (0.90–3.35)	1.49 (0.70–3.17)
Endocrine therapy	1	1.40 (1.16–1.68)	1.23 (1.00–1.50)	0.24 (0.03–1.86)	0.25 (0.03–2.20)	0.96 (0.66–1.41)	0.83 (0.54–1.26)
	2	1.03 (0.80–1.34)	0.84 (0.64–1.12)	N.E	N.E	1.02 (0.66–1.58)	0.81 (0.49–1.34)
	Pooled	1.09 (0.96–1.23)	0.95 (0.83–1.08)	1.26 (1.08–1.46)	1.07 (0.91–1.26)	0.21 (0.03–1.59)	0.25 (0.03–2.09)

^a^The model was adjusted for Arrhythmia (other than AF), Bleeding Stroke, Cancer, Heart Failure, Ischemic heart disease, Ischemic Stroke, Liver disease, Lung disease, Other Vascular disease, Renal disease, Valve disorder, Venous Thromboembolism, Insulins and Analogues, Antithrombotic Agents, Cardiac Therapy, Antihypertensives, Diuretics, Beta Blocking Agents, Calcium Channel Blockers, Agents Acting on the Renin-Angiotensin System, Lipid Modifying Agents, Sex Hormones and Modulators of the Genital System, Corticosteroids for Systemic Use, Endocrine Therapy and Year of birth

**Fig. 2:**
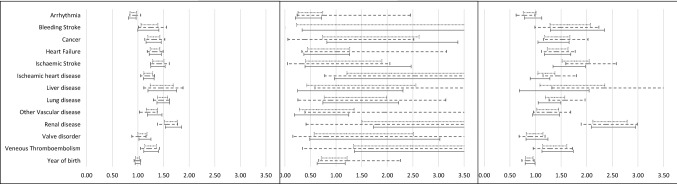
Odds ratios with 95% confidence intervals for comorbidities for each outcome, hospitalization, ICU admission and death, by COVID-19 wave and pooled. Solid line: wave 1, dashed line: wave 2, dotted line: pooled. The model was adjusted for Arrhythmia (other than AF), Bleeding Stroke, Cancer, Heart Failure, Ischemic heart disease, Ischemic Stroke, Liver disease, Lung disease, Other Vascular disease, Renal disease, Valve disorder, Venous Thromboembolism, Insulins and Analogues, Antithrombotic Agents, Cardiac Therapy, Antihypertensive, Diuretics, Beta Blocking Agents, Calcium Channel Blockers, Agents Acting on the Renin-Angiotensin System, Lipid Modifying Agents, Sex Hormones and Modulators of the Genital System, Corticosteroids for Systemic Use, Endocrine Therapy and Year of birth

Figure 2 displays data on comorbid covariates. Notably, arrythmia (other than AF) was associated with decreased odds ratios of hospitalization and ICU admission during the first wave; it was close to significant regarding mortality. In contrast, most other comorbid conditions were—as expected, and as previously reported [28]—related to increased risks of all three outcomes.

## Discussion

### Key findings

In this nested case–control study from the Stockholm Region the use of antithrombotic drugs was associated with lower risk of hospitalization, ICU admission and death during infection with SARS-CoV-2 in patients with arrythmias.

### Relationship to previous studies

We hypothesized that anticoagulation reduces risk of adverse outcomes, measured by risk of hospitalization, ICU admission and death, in COVID-19-patients. Similar assumptions have been tested by other research groups. However, evidence regarding medication with antithrombotic drugs before exposure- and admission for COVID-19 is plagued by an inability to properly adjust for confounding. Additionally, the field has a relative lack of large-scale investigations and high-resolution data. This triad of issues contributes to the confusion; findings with regards to pre-COVID-antithrombotic medication range from associations with higher mortality, via no discernable effects, to improved outcomes including lower mortality. Sub-analysis of the HOPE COVID-19 registry, a cohort of 1002 patients and 110 of which were on oral anticoagulation, showed higher mortality risk compared to propensity score matched patients [29]. In a single-center, retrospective observational study, from the emergency department of an Italian teaching hospital, 1407 patients over 65 years, with (9.6%) or without (90.4%) OACs were evaluated [30]. The authors report that crude hospital mortality rate was higher for medicated patients, but not so after multivariable adjustments. Similarly, an observational study from Poland demonstrated that pre-COVID-19-anticoagulation had no impact on middle-term mortality [31]. A nationwide pharmacoepidemiologic study from Sweden [10] assessed impact of antithrombotic medication on hospitalization and a composite outcome of ICU admission and death from February to May 2020. In a publication from 2020, Harenberg et al. notes that the number of patients with non-valvular AF and severe COVID-19 symptoms is low, which they suggest might be explained by a beneficial effect of prehospital oral anticoagulation and in-hospital heparinization [32].

In total, 360 patients were hospitalized for COVID-19 and 160 patients presented the composite outcome, but this did not differ significantly from the comparator group. This composite outcome is problematic, since ICU admission in Stockholm during the first wave was prone to triage/patient selection due to the massive surge of patients; described in detail in a recent publication [28]. Therefore, we speculate that the opposing results between waves for ICU admission with antithrombotic exposure might be explained by triaging. In contrast, mentioned in the introduction, the multinational Lean European Open Survey on SARS-CoV-2 infected patients (LEOSS) [9] analyzed 1433 patients, with 334 (23.3%) using OAC [9]. After adjustments, pre-existing OAC was associated with lower risk of death (OR 0.64), as non-recovery (OR 0.66) and a combined endpoint of death or thrombotic event (OR 0.71). A single-center study from Spain of 1612 subjects, found lower ICU admission rates for patients on anticoagulation therapy [33]. A nationwide cohort of 6637 hospitalized patients in Germany evaluated impact of oral anticoagulation (n = 1578) on clinical outcomes of COVID-19 [34]. The primary endpoint was a composite of all-cause mortality or need for invasive or non-invasive ventilation or extracorporeal membrane oxygenation. Even after propensity scoring, direct oral anticoagulants or vitamin-K antagonists, but not antiplatelet therapy, were significantly associated with improved clinical outcomes. In Italy, the multicenter GeroCovid observational study allowed for a closer evaluation of exclusively atrial fibrillation patients, with and without anticoagulation therapy [35]. Among these 171 patients, both vitamin K-antagonists and direct OACs, was associated with lower mortality. The Italian study, albeit much smaller, is similar to our study: using only patients with a diagnosis of atrial fibrillation.

### Significance of study findings

Arrythmias, including atrial fibrillation, are coupled with other more severe cardiac diseases and frequently with significant non-cardiac comorbidities [36]. This may explain why some studies on anticoagulation therapy fail to fully adjust or account for this confounding. Most researchers and clinicians do agree that AF patients should—like all patients with cardiac co-morbidities—be considered vulnerable, with increased risk of fatal outcome, prompting high-level clinical monitoring and treatment [37]. In line with this, a New York based cohort was evaluated using propensity scoring of patients with and without AF; demonstrating a hospital mortality of 54 vs 37% [38]. Systemic inflammation of epicardial fat is prevalent among AF patients. Speculation as to if this inflammatory state is amplified by COVID-19, leading to worse outcomes exist [39]. Therefore, it is noteworthy that we present Stockholm data indicating that arrythmias differs to most other comorbid conditions with regards to risk of death. Naturally, the prevalence of possibly protective co-medication may play a role. COVID-19 causes widespread thromboembolic complications [40], both in small and large vessels. Reducing these complications, in particular pulmonary embolism, may be one explanation to the favorable outcome seen in the present study.

### Strengths and weaknesses

This study has strengths and weaknesses. We have access to high resolution data on medications, co-morbid conditions, COVID-infections, hospital and ICU admission and date of death. This allowed us to create a large dataset using only AF-patients. Specifically, this was done to address issues of confounding by indication. The detailed data was paramount for the matching procedure as described in the methods. Moreover, it made it possible to adjust for confounding factors to an extent that is unusual. Weaknesses include an inability to know why some patients with atrial fibrillation were unmedicated. Reasons are likely spanning from a spectrum of non-compliant/non-health seeking behavior, often associated with elevated risks of adverse outcomes, to the polar opposite: low risk patients where their doctors’ choice was not to medicate, due to only intermittent AF. Therefore, untreated AF might be a source of unmeasured confounding. Moreover, one can never be sure that the prescribed and collected medication actually is used by every single subject. Another weakness has been mentioned; using ICU admission as an outcome during the first wave in Sweden is problematic. Data on ICU admission is of high quality, but the March and April 2020 surge of patients means that this outcome was distorted by patient selection issues: elderly patients with high illness severity, i.e., more comorbidities or co-medication, were often not admitted [28]. This is demonstrated in the tables, where especially co-medication was seen as “protective” during the first wave, but not during the second. Moreover, we could not use COVID-19 infection as a measure since testing was voluntary in Sweden. Finally, residual confounding cannot be ruled out.

## Conclusions

We conclude that the use of anticoagulation therapy among patients with COVID-19 and arrythmias is associated with lower risk of hospitalization and death. This data should significantly alter how medication and risk-reward is evaluated in these patients. If further COVID-variants emerge, or in the event of other infectious diseases with prothrombotic properties, this further emphasize the need for physicians to ensure compliance among these vulnerable patient groups, such as those with arrythmias.

## Supplementary Information

Below is the link to the electronic supplementary material.

Supplementary file1 (DOCX 21 KB)

Supplementary file2 (DOCX 16 KB)

## Declarations

### Data sharing

Data cannot be shared due to Swedish legislation concerning secrecy.

### Transparency statement

The lead author Max Bell affirms that this manuscript is an honest, accurate, and transparent account of the study being reported; that no important aspects of the study have been omitted; and that any discrepancies from the study as planned have been explained.

### Dissemination to participants and related patient and public communities

To be provided if accepted.
