# Supplementary material for: COVID-19 and comedications in atrial fibrillation—a case–control study in Stockholm
Source: Eur J Epidemiol. 2023 Jan 28;38(3):301–11. doi: 10.1007/s10654-023-00967-9 (PMC9883132; doi:10.1007/s10654-023-00967-9)
Supplement: Supplementary file 1 — Supplementary file1 (DOCX 21 KB) [file 10654_2023_967_MOESM1_ESM.docx]

Supplementary table 1: ATC codes used to identify exposures

| **Exposure group** | **ATC code** | **Substance** |
| --- | --- | --- |
| A10 | A10AB01 | Insulin (human) |
|  | A10AB04 | Insulin lispro |
|  | A10AB05 | Insulin aspart |
|  | A10AB06 | Insulin glulisine |
|  | A10AC01 | Insulin (human) |
|  | A10AD01 | Insulin (human) |
|  | A10AD04 | Insulin lispro |
|  | A10AD05 | Insulin aspart |
|  | A10AE01 | Insulin (human) |
|  | A10AE04 | Insulin glargine |
|  | A10AE05 | Insulin detemir |
|  | A10AE06 | Insulin degludec |
|  | A10AE54 | Insulin glargine and lixisenatide |
|  | A10AE56 | Insulin degludec and liraglutide |
|  | A10BA02 | Metformin |
|  | A10BB01 | Glibenclamide |
|  | A10BB03 | Tolbutamide |
|  | A10BB07 | Glipizide |
|  | A10BB12 | Glimepiride |
|  | A10BD03 | Metformin and rosiglitazone |
|  | A10BD05 | Metformin and pioglitazone |
|  | A10BD07 | Metformin and sitagliptin |
|  | A10BD08 | Metformin and vildagliptin |
|  | A10BD10 | Metformin and saxagliptin |
|  | A10BD11 | Metformin and linagliptin |
|  | A10BD15 | Metformin and dapagliflozin |
|  | A10BD20 | Metformin and empagliflozin |
|  | A10BD21 | Saxagliptin and dapagliflozin |
|  | A10BD23 | Metformin and ertugliflozin |
|  | A10BF01 | Acarbose |
|  | A10BG02 | Rosiglitazone |
|  | A10BG03 | Pioglitazone |
|  | A10BH01 | Sitagliptin |
|  | A10BH02 | Vildagliptin |
|  | A10BH03 | Saxagliptin |
|  | A10BH05 | Linagliptin |
|  | A10BJ01 | Exenatide |
|  | A10BJ02 | Liraglutide |
|  | A10BJ03 | Lixisenatide |
|  | A10BJ05 | Dulaglutide |
|  | A10BJ06 | Semaglutide |
|  | A10BK01 | Dapagliflozin |
|  | A10BK02 | Canagliflozin |
|  | A10BK03 | Empagliflozin |
|  | A10BK04 | Ertugliflozin |
|  | A10BX02 | Repaglinide |
|  | A10BX03 | Nateglinide |
| B01 | B01AA03 | Warfarin |
|  | B01AA04 | Phenprocoumon |
|  | B01AA07 | Acenocoumarol |
|  | B01AB01 | Heparin |
|  | B01AB04 | Dalteparin |
|  | B01AB05 | Enoxaparin |
|  | B01AB09 | Danaparoid |
|  | B01AB10 | Tinzaparin |
|  | B01AC04 | Clopidogrel |
|  | B01AC05 | Ticlopidine |
|  | B01AC06 | Acetylsalicylic acid |
|  | B01AC07 | Dipyridamole |
|  | B01AC09 | Epoprostenol |
|  | B01AC11 | Iloprost |
|  | B01AC21 | Treprostinil |
|  | B01AC22 | Prasugrel |
|  | B01AC23 | Cilostazol |
|  | B01AC24 | Ticagrelor |
|  | B01AC27 | Selexipag |
|  | B01AC30 | Combinations |
|  | B01AD02 | Alteplase |
|  | B01AE05 | Ximelagatran |
|  | B01AE07 | Dabigatran etexilate |
|  | B01AF01 | Rivaroxaban |
|  | B01AF02 | Apixaban |
|  | B01AF03 | Edoxaban |
|  | B01AX05 | Fondaparinux |
| C01 | C01AA04 | Digitoxin |
|  | C01AA05 | Digoxin |
|  | C01BA01 | Quinidine |
|  | C01BA03 | Disopyramide |
|  | C01BB02 | Mexiletine |
|  | C01BC03 | Propafenone |
|  | C01BC04 | Flecainide |
|  | C01BD01 | Amiodarone |
|  | C01BD07 | Dronedarone |
|  | C01CA01 | Etilefrine |
|  | C01CA07 | Dobutamine |
|  | C01CA17 | Midodrine |
|  | C01CA24 | Adrenaline |
|  | C01DA02 | Glyceryl trinitrate |
|  | C01DA08 | Isosorbide dinitrate |
|  | C01DA14 | Isosorbide mononitrate |
|  | C01EB09 | Ubidecarenone |
|  | C01EB15 | Trimetazidine |
|  | C01EB17 | Ivabradine |
| C02 | C02AB01 | Methyldopa (levorotatory) |
|  | C02AC01 | Clonidine |
|  | C02AC02 | Guanfacine |
|  | C02AC05 | Moxonidine |
|  | C02CA01 | Prazosin |
|  | C02CA04 | Doxazosin |
|  | C02DB02 | Hydralazine |
|  | C02DC01 | Minoxidil |
|  | C02KX01 | Bosentan |
|  | C02KX02 | Ambrisentan |
|  | C02KX04 | Macitentan |
|  | C02KX05 | Riociguat |
| C03 | C03AA01 | Bendroflumethiazide |
|  | C03AA03 | Hydrochlorothiazide |
|  | C03AB01 | Bendroflumethiazide and potassium |
|  | C03BA04 | Chlortalidone |
|  | C03BA08 | Metolazone |
|  | C03CA01 | Furosemide |
|  | C03CA02 | Bumetanide |
|  | C03CA04 | Torasemide |
|  | C03DA01 | Spironolactone |
|  | C03DA04 | Eplerenone |
|  | C03DB01 | Amiloride |
|  | C03EA01 | Hydrochlorothiazide and potassium-sparing agents |
|  | C03XA01 | Tolvaptan |
| C07 | C07AA03 | Pindolol |
|  | C07AA05 | Propranolol |
|  | C07AA06 | Timolol |
|  | C07AA07 | Sotalol |
|  | C07AB02 | Metoprolol |
|  | C07AB03 | Atenolol |
|  | C07AB07 | Bisoprolol |
|  | C07AB08 | Celiprolol |
|  | C07AB12 | Nebivolol |
|  | C07AG01 | Labetalol |
|  | C07AG02 | Carvedilol |
|  | C07FB02 | Metoprolol and felodipine |
| C08 | C08CA01 | Amlodipine |
|  | C08CA02 | Felodipine |
|  | C08CA03 | Isradipine |
|  | C08CA05 | Nifedipine |
|  | C08CA06 | Nimodipine |
|  | C08CA13 | Lercanidipine |
|  | C08DA01 | Verapamil |
|  | C08DB01 | Diltiazem |
| C09 | C09AA01 | Captopril |
|  | C09AA02 | Enalapril |
|  | C09AA03 | Lisinopril |
|  | C09AA04 | Perindopril |
|  | C09AA05 | Ramipril |
|  | C09AA06 | Quinapril |
|  | C09AA08 | Cilazapril |
|  | C09AA09 | Fosinopril |
|  | C09AA10 | Trandolapril |
|  | C09BA02 | Enalapril and diuretics |
|  | C09BA03 | Lisinopril and diuretics |
|  | C09BA05 | Ramipril and diuretics |
|  | C09BA06 | Quinapril and diuretics |
|  | C09BA08 | Cilazapril and diuretics |
|  | C09BB10 | Trandolapril and verapamil |
|  | C09CA01 | Losartan |
|  | C09CA02 | Eprosartan |
|  | C09CA03 | Valsartan |
|  | C09CA04 | Irbesartan |
|  | C09CA06 | Candesartan |
|  | C09CA07 | Telmisartan |
|  | C09DA01 | Losartan and diuretics |
|  | C09DA02 | Eprosartan and diuretics |
|  | C09DA03 | Valsartan and diuretics |
|  | C09DA04 | Irbesartan and diuretics |
|  | C09DA06 | Candesartan and diuretics |
|  | C09DA07 | Telmisartan and diuretics |
|  | C09DB01 | Valsartan and amlodipine |
|  | C09DX04 | Valsartan and sacubitril |
|  | C09XA02 | Aliskiren |
| C10 | C10AA01 | Simvastatin |
|  | C10AA03 | Pravastatin |
|  | C10AA04 | Fluvastatin |
|  | C10AA05 | Atorvastatin |
|  | C10AA07 | Rosuvastatin |
|  | C10AB02 | Bezafibrate |
|  | C10AB04 | Gemfibrozil |
|  | C10AB05 | Fenofibrate |
|  | C10AC01 | Colestyramine |
|  | C10AC02 | Colestipol |
|  | C10AC04 | Colesevelam |
|  | C10AD02 | Nicotinic acid |
|  | C10AD52 | Nicotinic acid, combinations |
|  | C10AX06 | Omega-3-triglycerides incl. other esters and acids |
|  | C10AX09 | Ezetimibe |
|  | C10AX13 | Evolocumab |
|  | C10AX14 | Alirocumab |
|  | C10BA02 | Simvastatin and ezetimibe |
|  | C10BA05 | Atorvastatin and ezetimibe |
| G03 | G03AA03 | Lynestrenol and ethinylestradiol |
|  | G03AA05 | Norethisterone and ethinylestradiol |
|  | G03AA07 | Levonorgestrel and ethinylestradiol |
|  | G03AA09 | Desogestrel and ethinylestradiol |
|  | G03AA11 | Norgestimate and ethinylestradiol |
|  | G03AA12 | Drospirenone and ethinylestradiol |
|  | G03AA13 | Norelgestromin and ethinylestradiol |
|  | G03AA14 | Nomegestrol and estradiol |
|  | G03AA16 | Dienogest and ethinylestradiol |
|  | G03AB03 | Levonorgestrel and ethinylestradiol |
|  | G03AB04 | Norethisterone and ethinylestradiol |
|  | G03AB05 | Desogestrel and ethinylestradiol |
|  | G03AB08 | Dienogest and estradiol |
|  | G03AC01 | Norethisterone |
|  | G03AC02 | Lynestrenol |
|  | G03AC03 | Levonorgestrel |
|  | G03AC06 | Medroxyprogesterone |
|  | G03AC08 | Etonogestrel |
|  | G03AC09 | Desogestrel |
|  | G03AC10 | Drospirenone |
|  | G03AD01 | Levonorgestrel |
|  | G03AD02 | Ulipristal |
|  | G03BA03 | Testosterone |
|  | G03BB02 | Androstanolone |
|  | G03CA03 | Estradiol |
|  | G03CA04 | Estriol |
|  | G03CA57 | Conjugated estrogens |
|  | G03CC05 | Diethylstilbestrol |
|  | G03CX01 | Tibolone |
|  | G03DA02 | Medroxyprogesterone |
|  | G03DA04 | Progesterone |
|  | G03DB01 | Dydrogesterone |
|  | G03DB08 | Dienogest |
|  | G03DC02 | Norethisterone |
|  | G03DC03 | Lynestrenol |
|  | G03FA01 | Norethisterone and estrogen |
|  | G03FA12 | Medroxyprogesterone and estrogen |
|  | G03FA14 | Dydrogesterone and estrogen |
|  | G03FA15 | Dienogest and estrogen |
|  | G03FA17 | Drospirenone and estrogen |
|  | G03FB05 | Norethisterone and estrogen |
|  | G03FB06 | Medroxyprogesterone and estrogen |
|  | G03FB08 | Dydrogesterone and estrogen |
|  | G03FB09 | Levonorgestrel and estrogen |
|  | G03GA01 | Chorionic gonadotrophin |
|  | G03GA02 | Human menopausal gonadotrophin |
|  | G03GA04 | Urofollitropin |
|  | G03GA05 | Follitropin alfa |
|  | G03GA06 | Follitropin beta |
|  | G03GA07 | Lutropin alfa |
|  | G03GA08 | Choriogonadotropin alfa |
|  | G03GA09 | Corifollitropin alfa |
|  | G03GB02 | Clomifene |
|  | G03HA01 | Cyproterone |
|  | G03HB01 | Cyproterone and estrogen |
|  | G03XA01 | Danazol |
|  | G03XB01 | Mifepristone |
|  | G03XB02 | Ulipristal |
|  | G03XC01 | Raloxifene |
|  | G03XX01 | Prasterone |
| H02 | H02AA02 | Fludrocortisone |
|  | H02AB01 | Betamethasone |
|  | H02AB02 | Dexamethasone |
|  | H02AB04 | Methylprednisolone |
|  | H02AB06 | Prednisolone |
|  | H02AB07 | Prednisone |
|  | H02AB08 | Triamcinolone |
|  | H02AB09 | Hydrocortisone |
|  | H02AB10 | Cortisone |
| L02 | L02AA02 | Polyestradiol phosphate |
|  | L02AB01 | Megestrol |
|  | L02AB02 | Medroxyprogesterone |
|  | L02AE01 | Buserelin |
|  | L02AE02 | Leuprorelin |
|  | L02AE03 | Goserelin |
|  | L02AE04 | Triptorelin |
|  | L02AE05 | Histrelin |
|  | L02BA01 | Tamoxifen |
|  | L02BA02 | Toremifene |
|  | L02BA03 | Fulvestrant |
|  | L02BB01 | Flutamide |
|  | L02BB02 | Nilutamide |
|  | L02BB03 | Bicalutamide |
|  | L02BB04 | Enzalutamide |
|  | L02BB06 | Darolutamide |
|  | L02BG03 | Anastrozole |
|  | L02BG04 | Letrozole |
|  | L02BG06 | Exemestane |
|  | L02BX02 | Degarelix |
|  | L02BX03 | Abiraterone |
