# Supplementary material for: COVID-19 and comedications in atrial fibrillation—a case–control study in Stockholm
Source: Eur J Epidemiol. 2023 Jan 28;38(3):301–11. doi: 10.1007/s10654-023-00967-9 (PMC9883132; doi:10.1007/s10654-023-00967-9)
Supplement: Supplementary file 2 — Supplementary file2 (DOCX 16 KB) [file 10654_2023_967_MOESM2_ESM.docx]

Supplementary table 2: Diagnosis codes (ICD-10) and procedure codes (NCSP/KVÅ) used to identify comorbidities and outcomes.

| **Variable** | **Code** | |
| --- | --- | --- |
| **Comorbidities (10-year look-back)** | **ICD-10-SE code** | **Procedure code (NCSP/KVÅ)** |
| IHD=Ischemic heart disease | I20.n, I21.n, I22.n, I23.n, I24.n, I25.n | FNAnn, FNBnn, FNCnn, FNDnn, FNEnn, FNFnn, FNGnn, DF009, DF019, DF020 |
| HF=Heart failure/cardiomyopathy | I11.0, I13.0, I13.2, I13.3, I13.4, I13.5, I13.6, I13.7, I13.8, I13.9, I42.n, I43.n, I50.n, J81.9 |  |
| VD=Valve disorder | I05.1, I05.8, I05.9, I06.n, I07.n, I08.n, I34.0, I34.1, I34.3, I34.4, I34.5, I34.6, I34.7, I348, I34.9, I35.n, I36.n, I37.n |  |
| ISTR=Ischemic stroke/TIA/systemic thromboembolism | I63.n, I69.3, I74.n |  |
| BSTR=Hemorrhagic/unspecified stroke | I60.n, I61.n, I62.n, I64.9, I69.0, I69.1, I69.2 |  |
| OVD=Other vascular disease | I70.n, I71.n, I72.n, I73.n, I75.n, I76.n, I77.n, I79.0, I79.1, I79.2, I79.3, I79.4, I79.5, I79.6, I79.7, I79.9 |  |
| ARR=Arrhythmia (other than AF/flutter) | I44.1, I44.2, I45.3, I45.6, I46.n, I47.n, I49.n |  |
| LUD=Lung disease | I27.n, J40.n, J41.n, J42.n, J43.n, J44.n, J45.n, J46.9, J47.n, J60.n, J61.n, J62.n, J63.n, J64.n, J65.n, J66.n, J67.n, J68.4, J70.1, J70.3, J84.n, J92.n, J96.1, J98.2, J98.3 |  |
| RD=Renal disease | I12.n, I13.1, N17.n, N18.n, N19.n | KASnn |
| LID=Liver disease | B18.n, K70.n, K71.n, K72.n, K73.n, K74.n, K75.n, K76.n, K77.n | JJCnn |
| VTE=Venous thromboembolism | I26.n, I63.6, I67.6, I80.n, I81.n, I82.n, I87.0 |  |
| CAN=Malignancy (1-year look-back) | C00.n – C97.n |  |
| **COVID-19 Outcome** | **ICD-10-SE code** | **Procedure code (NCSP/KVÅ)** |
| Hospitalization | U07.1, U07.2, U10.9 | ZV100, AV097, DV091, GD001, XS100, DG015, DG028, DV028, QD014 |
| Death | U07.1, U07.2, U10.9 |  |
